# Development of Peptide Targeted PLGA-PEGylated Nanoparticles Loading Licochalcone-A for Ocular Inflammation

**DOI:** 10.3390/pharmaceutics14020285

**Published:** 2022-01-26

**Authors:** Ruth Galindo, Elena Sánchez-López, María José Gómara, Marta Espina, Miren Ettcheto, Amanda Cano, Isabel Haro, Antoni Camins, María Luisa García

**Affiliations:** 1 Department of Pharmacy, Pharmaceutical Technology and Physical Chemistry, Faculty of Pharmacy and Food Sciences, University of Barcelona, 08028 Barcelona, Spain; ruth.galindo@ub.edu (R.G.); m.espina@ub.edu (M.E.); acanofernandez@ub.edu (A.C.); marisagarcia@ub.edu (M.L.G.); 2 Unit of Synthesis and Biomedical Applications of Peptides, IQAC-CSIC, 08034 Barcelona, Spain; mjgqpp@cid.csic.es (M.J.G.); isabel.haro@iqac.csic.es (I.H.); 3 Institute of Nanoscience and Nanotechnology (IN2UB), University of Barcelona, 08028 Barcelona, Spain; 4 Biomedical Research Networking Centre in Neurodegenerative Diseases (CIBERNED), 28031 Madrid, Spain; mirenettcheto@ub.edu (M.E.); camins@ub.edu (A.C.); 5 Department of Pharmacology and Therapeutic Chemistry, Faculty of Pharmacy, University of Barcelona, 08028 Barcelona, Spain

**Keywords:** Licochalcone-A, nanoparticles, ocular inflammation, cell-penetrating peptides, PLGA

## Abstract

Licochalcone-A is a natural compound with anti-inflammatory properties. However, it possesses low water solubility, making its application for the treatment of ocular inflammation difficult. To overcome this drawback, biodegradable nanoparticles incorporating Licochalcone-A have been developed. Additionally, to avoid fast clearance and increase cellular internalization into the ocular tissues, PLGA nanoparticles have been functionalized using PEG and cell penetrating peptides (Tet-1 and B6). To optimize the formulations, a factorial design was carried out and short-term stability of the nanoparticles was studied. Moreover, morphology was also observed by transmission electron microcopy and in vitro drug release was carried out. Ocular tolerance of the formulations was ensured in vitro and in vivo and anti-inflammatory therapeutic efficacy was also assessed. Surface functionalized nanoparticles loading Licochalcone-A were developed with an average size below 200 nm, a positive surface charge, and a monodisperse population. The formulations were non-irritant and showed a prolonged Licochalcone-A release. Despite the fact that both Licochalcone-A Tet-1 and B6 functionalized nanoparticles demonstrated to be suitable for the treatment of ocular inflammation, B6 targeted nanoparticles provided greater therapeutic efficacy in in vivo assays.

## 1. Introduction

One of the most prevalent conditions in ophthalmology is ocular inflammation. This inflammation constitutes the local response of ocular tissues and annexes against an external or internal insult. Moreover, this process may be able to produce irreversible damage to the ocular function [1]. The inflammation process triggers the production of growth factors and cytokines and stimulates the enzyme phospholipase A2 (PLA2), which leads to the synthesis of eicosanoids from a phospholipid found in cell membranes, arachidonic acid (AA). AA can stimulate the production of pro-inflammatory cytokines and initiate apoptosis. Moreover, AA-derived eicosanoids regulate immunopathological and inflammatory processes through different physiological responses, vascular homeostasis, and platelet aggregation [2].

For the treatment of ocular inflammation, non-steroidal anti-inflammatory drugs as well as corticoids are currently used. However, both cause several adverse effects [3]. In this area, research on novel approaches avoiding side effects constitutes an unmet medical need. In this sense, Licochalcone-A (Lico-A) is a characteristic chalcone isolated from the root of *Glycyrrhiza inflata*, which has been used in traditional medicine to treat various disorders such as gastric ulcer, bronchial asthma, and inflammation [4]. The biological properties of Lico-A include antiparasitic, antiproliferative, antitumoral, antimicrobial, antiviral, osteogenic, immunomodulatory, and anti-inflammatory activities [5,6]. Moreover, Lico-A exhibits a carrageenan-induced anti-inflammatory effect in a murine model [6]. Despite the suitable properties of Lico-A against ocular inflammation, its solubility in water is extremely low, making its topical administration as conventional eye-drops difficult [7,8]. Moreover, it has also been reported that less than 5% of conventional ocular formulations are able reach inner ocular tissues [9].

In order to overcome these problems, nanotechnological drug delivery constitutes a suitable approach for active compounds encapsulation and their delivery into target sites [10,11,12]. Controlled administration of drugs, specially at the ocular level, can offer enormous benefits, including maximizing drug efficacy, minimizing side effects, and improving patient compliance [13,14].

Nanocarriers, such as nanoparticles (NPs), with sizes ranging between 10 and 1000 nm are of particular interest as drug delivery platforms for treating ocular diseases [15]. Among several carriers, biodegradable polymeric nanoparticles provide several advantages, such as protect the drug from inactivation by enzymes present in the tear film or corneal epithelium, facilitate transcorneal penetration, prolong its stability in the precorneal area, and avoid adverse effects in other organs [1]. Among them, biodegradable polymers such as poly (lactic-co-glycolic acid) (PLGA) have been widely used to produce NPs due to their biodegradability, biocompatibility, and mechanical resistance. In addition, PLGA is also approved by the Food and Drug Administration (FDA) for its use in various biomedical applications, such as absorbable sutures and implants, as well as for the development of sustained release systems [16,17]. Furthermore, compared to natural polymers, these synthetic polymers show greater reproducibility, are easier to formulate, and allow the control and prediction of degradation kinetics [18].

In addition, PLGA nanocarriers can be functionalized on their surface and targeted towards specific cells or ocular structures, increasing their transport across physiological barriers with a sustained and controlled release over time and reducing adverse effects of drugs [19,20]. PLGA nanocarriers minimize drug degradation and increase its half-life inside the target organ thus improving its efficacy and safety [21]. However, PLGA suffers from fast elimination in body tissues [22]. In order to overcome this problem, PEGylation is one of the most suitable approaches. Therefore, encapsulation of active compounds into poly(ethylene glycol) (PEG)-coated NPs offers the possibility to increase the transport across biological layers and increase drug protection against degradation [23].

Furthermore, during recent years, functionalization of NPs using cell-penetrating peptides (CPPs) has emerged as a new technology to facilitate the internalization of macromolecules and drugs in ocular cells, constituting a revolutionary approach due to their high transduction efficiency and low cytotoxicity [24,25]. CPPs are peptide sequences between 5 and 30 amino acids (AA) long with the ability to be internalized in the cell [26,27]. Among the different types of CPPs, cationic CPPs possess a highly positive surface charge due to their sequence rich in arginine residues (Arg) [28]. In this area, one of the most novel approaches is peptide functionalization of PLGA NPs by coupling selected CPPs to the NPs surface increasing cellular internalization. Among several peptides, Tet-1 and B6 were selected as suitable candidates for ocular drug delivery (Figure S1). Tet-1 is a 12-AA peptide, with the sequence HLNILSTLWKYR, which has the binding characteristics of tetanus toxin, can interact specifically with motor neurons, and is capable of retrograde delivery in the neuronal tissues due to its affinity to ganglioside GT1B receptor [29,30,31]. Therefore, Tet-1 peptide might increase cellular internalization also on the ocular tissues due to the presence of ganglioside receptors in this organ [30,31,32,33]. Moreover, a peptide showing high affinity to transferrin receptor (GHKAKGPRK, namely B6) has been described to increase transport across the BBB [20,34]. Since ocular tissues, and more specifically, corneal layers, have been reported to possess transferrin receptors, hereby, we postulate that B6 targeting may also be useful for ocular drug delivery [35,36,37]. To our knowledge, to date no attempt has been carried out using Tet-1 or B6 CPPs for surface functionalization aimed at ocular drug delivery.

Therefore, the main goal of this study was the entrapment of Lico-A inside surface functionalized Tet-1 or B6 PLGA-PEG NPs for ocular administration. PLGA NPs, synthesized by the solvent displacement method, were optimized using the design of experiments (DoE) approach, analyzing their physicochemical properties and stability. Subsequently, the study of drug release, ocular tolerance, and in vivo therapeutic efficacy of Lico-A PLGA NPs, Lico-A PLGA-PEG-Tet1 NPs, and Lico-A PLGA-PEG-B6 NPs was carried out.

## 2. Materials and Methods

### 2.1. Materials

The active compound Lico-A was obtained from Amadis Chemical (Hangzhou, China) and the polymer PLGA 50:50 Resomer^®^ RG 503 H, 34 KD, from Boehringer Ingelheim (Ingelheim, Germany). Maleimide-PEG-NH_2_ (5KDa) was from Jenkem (Beijing, China). NovaSyn TGR resin and 9-fluorenyl-methoxycarbonyl (Fmoc) protected amino acids were purchased from Novabiochem (Merck Millipore, Merck KGaA, Darmstadt, Germany). Peptide-synthesis-grade dimethylformamide (DMF) and trifluroacetic acid (TFA) were obtained from Scharlau (Barcelona, Spain). HPLC-grade acetonitrile and acetone were purchased from Fisher Scientific (Loughborough, UK). The coupling reagent, 2-(1H-7-azabenzotriazole-1-yl)-1,1,3,3- tetra-methyluronium hexafluorophosphate methanaminium (HATU) was from Genscript (Piscataway, NJ, USA). Diisopropylethylamine (DIPEA), piperidine, triisopropylsilane (TIS), 2-mercaptoethanol, *N*-(3-dimethylaminopropyl)-*N’*-ethylcarbodiimide hydrochloride (EDC), *N*-hydroxysuccinimide (NHS), dimethyl sulfoxide (DMSO), dimethyl sulfoxide-d6 (DMSO-d6), and sodium arachidonic acid (SA) were purchased from Sigma-Aldrich (Merck KGaA, Darmstadt, Germany). Methanol and diethyl ether were purchased from Merck (KGaA, Darmstadt, Germany). Water filtered through the Millipore^®^ MilliQ system was used for all experiments and the other chemicals and reagents used in the study were of analytical grade.

### 2.2. Preparation of Licochalcone-A PLGA Nanoparticles

For the elaboration of Lico-A PLGA NPs, the solvent displacement method was used [38,39]. Briefly, this technique consists of the preparation of two phases, namely organic and aqueous phases. First, 80 mg of PLGA Resomer^®^ RG 503 H and 10 mg of the drug Lico-A were dissolved in 5 mL of acetone (volatile organic solvent). Subsequently, this solution was dispersed dropwise, under moderate mechanical stirring, in 10 mL of the aqueous phase containing Tween 80; this process was carried out at room temperature. Afterwards, the organic solvent was evaporated from the suspension under reduced pressure using a rotary evaporator.

### 2.3. Physicochemical Characterization of Licochalcone-A PLGA Nanoparticles

In order to measure the physicochemical properties of Lico-A PLGA NPs, average size (Z_av_), polydispersity index (PI), zeta potential (ZP), and entrapment efficiency (EE) were determined.

Z_av_ and PI were placed in disposable cells (Malvern Instruments) and measured by photon correlation spectroscopy (PCS), after a 1:10 dilution with MilliQ^®^water, using the Zetasizer Nano ZS (Malvern Instruments, Malvern, UK) at 25 °C. The surface charge of the particles was evaluated by means of ZP and determined by laser-Doppler electrophoresis with the M3 PALS system in a Zetasizer Nano ZS (Malvern Instruments, Malvern, UK) [29].

EE was determined indirectly by measuring the non-entrapped Lico-A. The non-loaded drug was separated from NPs by ultracentrifugation at 4 °C and 25,000 rpm for 25 min, using a Beckman Optima^®^ Ultracentrifuge (Indianapolis, IN, USA), and the EE was evaluated according to Equation (1) [30].

(1) $$\mathrm{EE}\;(\%)=\frac{\mathrm{total}\;\mathrm{amount}\;\mathrm{of}\;\mathrm{Lico}-A\;-\;\mathrm{free}\;\mathrm{amount}\;\mathrm{of}\;\mathrm{Lico}-A\;}{\begin{array}{c} \mathrm{total}\;\mathrm{amount}\;\mathrm{of}\;\mathrm{Lico}-A \end{array}}\;\times \;100\;$$

Quantification of Lico-A in the aqueous phase was performed by reverse-phase high performance liquid chromatography (RP-HPLC) using a method described elsewhere [40,41]. HPLC Waters 2695 separation module (Waters, Milford, MA, USA) and a Kromasil^®^ C18 column (5 µm, 150 × 4.6 mm) (Teknokroma Analítica, Barcelona, Spain) were employed. Moreover, two mobile phases were used consisting of a water phase containing 0.2% acetic acid and an organic phase consisting of acetonitrile. A gradient was applied at 1.0 mL·min^−1^ (during the first 10 min an initial 35% mobile phase B was used until 100% mobile phase B, maintaining this percentage for 7 min more, and after that it was progressively modified for 5 min until 100% of mobile phase A). For the calibration curve, a concentration range from 10 to 100 µg·mL^−1^ was used. Agilent 1260 Infinity Variable Wavelength Detector VL standard version at a wavelength of 254 nm was utilized to identify Lico-A and data were handled using Empower 3^®^ Software. Limit of detection (LOD) and limit of quantification (LOQ) were calculated as described elsewhere being 2.48 and 8.27 µg·mL^−1^ [42].

### 2.4. Optimization of Licochalcone-A PLGA Nanoparticles

Optimization of Lico-A PLGA NPs was carried out prior to CPP addition by means of the Design of Experiments (DoE) approach. A 2^3^ central composite factorial design was carried out (Table 1) using StatGraphics Centurion XV, to analyze the effects of the independent variables over the dependent variables [43]. A total of 16 experiments were carried out by triplicate, with varying concentrations of drug (Lico-A), surfactant (Tween 80), and polymer. The effects of these variables on the dependent variables Z_av_, PI, ZP, and EE were studied.

### 2.5. Short-Term Stability of Licochalcone-A PLGA Nanoparticles

The stability of Lico-A PLGA NPs stored at 4, 25, and 38 °C was studied by analyzing light backscattering (BS) profiles by means of Turbiscan^®^ Lab (Formulaction, Toulouse, France) [44]. For this purpose, 20 mL of Lico-A PLGA NPs were introduced into a glass measurement cell. The light source was a pulsed near infrared light source (λ = 880 nm) and it was detected by a BS detector at an angle of 45° from the incident beam. BS data were obtained scanning the sample every hour during 24 h. The mean results of each month were plotted and BS profiles were compared monthly at each temperature.

### 2.6. Synthesis of Cell Penetrating Peptides

CPPs, Tet-1, and B6 (Figure S1) were selected based on their high affinity for cell receptors and were synthesized by manual solid phase synthesis following a 9-fluorenylmethoxycarbonyl/tbutyl (Fmoc/tBut) orthogonal protection strategy [45]. The C-terminal amino acid (AA) remains anchored to a resin (NovaSyn TGR 0.19 meq/g for Tet-1 and 0.22 meq/g for peptide B6) bound by its carboxyl group while the peptide is synthesized by elongating the chain at the N-terminal end. Amino acid side-chain protection was carried out using the following compounds: triphenylmethyl (Trt) for asparagine and histidine; tert-butyl (tBu) for serine, threonine, and tyrosine; 2,2,5,7,8-pentamethyl-chroman-6-sulfonyl (Pmc) for arginine and tert-butoxycarbonyl (Boc) for lysine and tryptophan. A residue of Fmoc-S-triphenylmethyl-L-cysteine (Fmoc-Cys(Trt)-OH) was incorporated at the N-terminal end of both peptides, Tet-1 and B6, for their conjugation to the polymer. Sequential incorporation of the Fmoc-L-amino acids is accomplished through a series of coupling and deprotection steps. The coupling reactions were performed using three-fold molar excesses of the amino acid derivatives activated by treatment with HATU and DIPEA throughout the synthesis. The Fmoc deprotection was accomplished twice with 20% (*v*/*v*) piperidine in DMF for 10 min. All coupling and deprotection steps were checked by the ninhydrin test for primary amines, or the chloranil test for secondary amino groups. Finally, peptides were cleaved from the solid support by means of treatment with TFA/TIS/ H_2_O/2-mercaptoethanol (94:2.5:2.5:1) (*v*/*v*) for 5 h. The TFA was evaporated under N_2_ flow. Diethyl ether was added to precipitate the crude peptides, which were isolated by centrifugation. The precipitates were dissolved in acetic acid 10%, frozen in a dry ice/acetone bath, and lyophilized. The peptides were stored in an argon environment to avoid their possible oxidation. Peptides were characterized by Ultra performance Liquid Chromatography–Mass Spectrometry (UPLC-MS) on Waters ACQUITY UPLC (Waters Corporation, Mildford, MA, USA) with the column ACQUITY UPLC BEH C18 (RP, 2.1 × 100 μm, particle size 1.7 μm) with both a UV-Vis detector and an electrospray ionization mass spectrometer (ESI-MS) Waters LCT Premier XE (Micromass Waters, Milford, MA, USA).

### 2.7. Conjugation of PEG and Cell Penetrating Peptides (CPPs) to the Polymer and Preparation of Lico-A PLGA-PEG-CPP NPs

**Figure 1:**
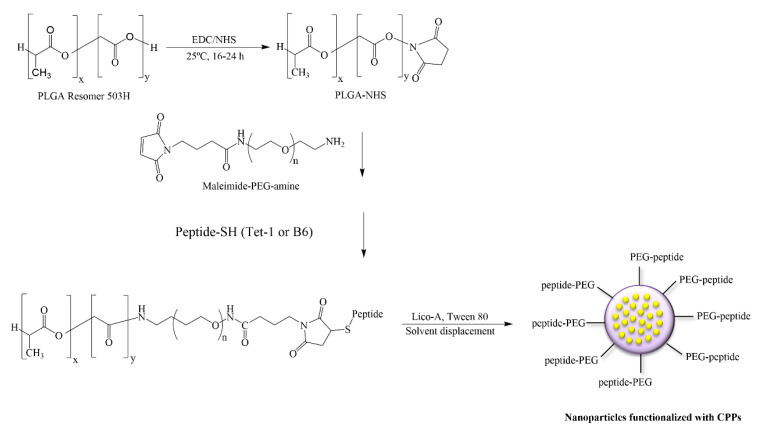
Conjugation of PEG and cell penetrating peptides (CPPs) to PLGA and preparation of Lico-A PLGA-PEG-CPP NPs.

In order to functionalize PLGA (Figure 1), first the PLGA-NHS polymer was synthesized, for which the equivalent of 32.3 μmol of PLGA 50:50 Resomer^®^ RG 503 H (inherent viscosity 0.32–0.44 dL/g) was weighed out and dissolved in 2 mL of chloroform. Subsequently, 234.6 µmol of NHS and the same equivalents of EDC were added and the mixture was left to react for between 16 and 24 h under continuous mechanical stirring in a hermetically sealed glass vial at room temperature. Once the PLGA-NHS was obtained, it was precipitated with cold diethyl ether and centrifuged at 4000 rpm for 10 min at room temperature. After removing the supernatant, the pellet was redissolved in 2 mL of chloroform and precipitated with cold diethyl ether. This dissolution–precipitation process was carried out in triplicate. The PLGA-NHS polymer obtained was dried using nitrogen gas (N_2_) and lyophilized.

Subsequently the activated polymer was conjugated with maleimide-PEG-NH_2_ and DIEA was used as the activator. The obtained PLGA-PEG-maleimide copolymer was lyophilized and stored at −20 °C.

The conjugation of the selected peptides to the PLGA-PEG-maleimide polymer was carried out by dissolving 2.7 μmol of each CPP in 250 μL of acetonitrile/DMF and was added later to a solution of 100 mg of dissolved PLGA-PEG-maleimide in 1mL of chloroform. The reaction was left for 16–24 h under magnetic stirring at room temperature. The product was precipitated with 1 mL of cold ether:methanol (50:50) and centrifuged at 10,000 rpm for 10 min. The supernatant was discarded, and the product was redissolved in 0.5 mL of chloroform. This cycle was repeated two additional times [11]. The final product was lyophilized and stored at −20 °C. Conjugation evaluation was carried out by proton nuclear magnetic resonance (1H-NMR) as previously described [19]. The PLGA-PEG-maleimide was dissolved in deuterated chloroform and the PLGA-PEG-peptide in dimethyl sulfoxide (DMSO)-d6. The spectrum was recorded at 298 K on a Varian Inova 400 MHz spectrometer (Agilent Technologies, Santa Clara, CA, USA). PLGA-PEG-CPP NPs containing Lico-A were prepared by the solvent displacement method as described in Section 2.2. The physicochemical properties of Lico-A PLGA-PEG-CPP NPs were determined as described in Section 2.3.

### 2.8. Transmission Electron Microscopy of Licochalcone-A Functionalized Nanoparticles

Morphologies of Lico-A PLGA NPs, Lico-A -PLGA-PEG-Tet1 NPs, and Lico-A PLGA-PEG-B6 NPs were studied by transmission electron microscopy (TEM) on a JEOL 1010 microscope (Akishima, Japan). To visualize the NPs, samples were previously diluted (1:5) and the Holey Carbon grids were activated with UV light. Samples were placed on the grid surface and negative staining was performed with uranyl acetate (2%) [46].

### 2.9. Drug Release of Licochalcone-A Functionalized Nanoparticles

The in vitro release profiles of Lico-A PLGA NPs, Lico-A PLGA-PEG-Tet1 NPs, and Lico-A PLGA-PEG-B6 NPs from the polymeric matrix were determined by bulk-equilibrium direct dialysis [47]. Briefly, 9 mL of Lico-A NPs were placed in a dialysis bag (Medicell International Ltd. MWCO 12–14,000) and dialyzed against 150 mL of release medium. In order to accomplish sink conditions, release medium was composed of ethanol–water 75:25, at 32 °C (temperature of corneal surface) and 0.3 mL of samples were withdrawn at regular time intervals during 24 h [48]. Lico-A content was analyzed by HPLC as previously described. Moreover, the total volume of release medium was kept constant by replacement with fresh medium throughout the experiment.

### 2.10. Ocular Tolerance of Licochalcone-A Functionalized Nanoparticles

#### 2.10.1. In Vitro Ocular Tolerance

In order to assess the in vitro ocular tolerance, the HET-CAM (hen’s egg–chorioallantoic membrane) test was developed as described in the INVITTOX protocol No. 15 [49,50]. This method is based on the observation of the irritating effects (hemorrhage, vasoconstriction, and coagulation) that may appear during the first five minutes after the application of 300 μL of the studied formulation on the chorioallantoic membrane (CAM) of a 10-day embryonated egg. These eggs were kept at a temperature of 12 ± 1 °C for at least 24 h before placing them in the incubator with controlled temperature (37.8 °C) and humidity (50–60%) during the days of incubation [46]. Free Lico-A (dissolved in DMF:PEG 400:MQ Water; 1:49:50), Lico-A PLGA NPs, and Lico-A PLGA-PEG-CPP NPs were evaluated. Controls were created using SDS 1% (positive control for slow irritation), NaOH 0.1 N (positive control for rapid irritation), and NaCl 0.9% (negative control).

Data were analyzed by calculating the ocular irritation index (OII) by applying Equation (2) (*n* = 3/group).

(2) $$\mathrm{OII}=\frac{301-H}{300}\;\times \;5+\frac{301-V}{300}\;\times \;7+\;\frac{301-C}{300}\;\times \;9$$

where *H* is the time in seconds in which the hemorrhage appears; *V* is the time in seconds in which vasoconstriction appears; *C* is the time in seconds in which coagulation appears.

According to the OII score, the products were classified into four categories as described elsewhere [44].

#### 2.10.2. In Vivo Ocular Tolerance

In order to confirm the in vitro results, the primary eye irritation test of Draize et al. was used [46,51]. Young adult male albino New Zealand rabbits with an average weight of 2.5 kg housed in individual cages were used. Animals were maintained in controlled temperature (17–23 °C) and relative humidity (60–80%) conditions with food and water supplemented ad libitum. This test was carried out in accordance with the Ethical Committee for Animal Experimentation of the University of Barcelona and current legislation (Decree 214/97, Gencat). The products Lico-A PLGA NPs, Lico-A PLGA-PEG-Tet-1 NPs, and Lico-A PLGA-PEG-B6 NPs were evaluated. Each product (50 µL) was instilled in the conjunctival sac of the right eye and a gentle massage was applied to ensure correct circulation of the sample. Readings were carried out 30 min after the application of the sample, using the left eye as a negative control (*n* = 3/group). OII was determined as previously described elsewhere [52].

### 2.11. Anti-Inflammatory Therapeutic Efficacy of Licochalcone-A Functionalized Nanoparticles

The ability of the developed formulations to revert to ocular inflammation was demonstrated in New Zealand rabbits. Therefore, Lico-A PLGA NPs, Lico-A PLGA-PEG-Tet-1 NPs, and Lico-A PLGA-PEG-B6 NPs were evaluated in vivo in New Zealand rabbits [52].

In order to induce ocular inflammation, 50 µL of SA 0.5% (*w*/*v*) dissolved in PBS (pH 7.4) were instilled in the right eye. After 30 min of the induction of inflammation, 50 µL of each formulation (Lico-A PLGA NPs, Lico-A PLGA-PEG-Tet-1 NPs, and Lico-A PLGA-PEG-B6 NPs or isotonic saline serum) were instilled in the conjunctival sac of the right eye, using the left eye as a control. Inflammation was evaluated 30 min after instillation of the formulations, and then at 60, 90, 120, 150, 180, and 210 min, according to a modified Draize scoring system [53,54]. Ocular inflammation score was calculated and expressed as mean ± SD. Moreover, inhibition inflammation % was also calculated according to Equation (3):

(3) $$\mathrm{Inflammation}\;\mathrm{inhibition}\;(\%)=\frac{C-T}{C}\;\times \;100$$

C = saline serum ocular inflammation (control group)

T = treated group ocular inflammation

### 2.12. Statistical Analysis

Statistical analyses were performed by using one-way ANOVA with Tukey post hoc test. All analyzed data were presented as mean ± SD. GraphPad Prism^®^ 6.01 software was used to analyze the data.

## 3. Results and Discussion

### 3.1. Optimization and Characterization of Licochalcone-A PLGA NPs

In order to optimize the formulation, Lico-A PLGA NPs were prepared by solvent displacement technique and optimized by DoE, analyzing the effect of independent variables (drug, surfactant, and polymer concentrations) on the dependent variables (Z_av_, PI, ZP, and EE). The results obtained for different formulations of the central composite factorial design can be observed in Table 2.

**Figure 2:**
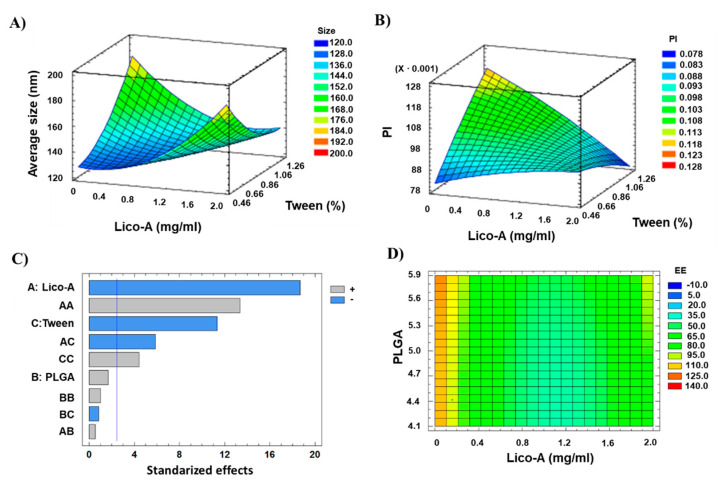
Design of experimental results. (**A**) Surface response plot for Z_av_, (**B**) Surface response plot for PI, (**C**) Pareto chart for EE (A: Lico-A concentration; B: PLGA concentration; C: Tween concentration; vertical blue line indicates significant effect), (**D**) Contoured surface response plot for EE.

Data obtained was analyzed in order to observe the trends that Lico-A PLGA NPs follow. Regarding the Z_av_, it can be observed that low Lico-A concentrations are able to provide smaller NPs size (Figure 2A). Moreover, high PLGA amounts tend to slightly increase NPs Z_av_ (Figure S2). This fact correlates with previous studies carried out using polymeric NPs where increased PLGA concentration causes higher Z_av_ [43]. Moreover, Z_av_ varies when low surfactant concentrations are used observing that at high surfactant amounts lower Z_av_ is obtained. Importantly, even with low surfactant amounts, all the formulations obtained showed Z_av_ below 200 nm. Moreover, increased amounts of Tween 80 favor lower PI values and, as observed for Z_av_, high Lico-A amounts tend to increase the PI (Figure 2B). Therefore, medium-low Lico-A concentrations will be used in order to reduce PI. Moreover, as can be observed in the Pareto chart, low amounts of Tween 80 also favor the EE (Figure 2C). Concerning this parameter, low Lico-A values tend to increase the EE (*p* < 0.05) (Figure 2D). This might be due to the fact that it is easier to encapsulate low amounts of drug, thus obtaining high EE values.

After analyzing the trends obtained and due to the fact that even at high PLGA amounts all Lico-A loaded NPs showed average sizes below 200 nm, the optimized formulation was developed and characterized containing 1 mg/mL of Lico-A, 8 mg/mL of PLGA, and 0.4% Tween. The physicochemical properties of the optimized NPs were suitable for ocular drug delivery since Z_av_ (163.81 ± 2.29) was below 200 nm, PI (0.075 ± 0.010) corresponded to monodisperse particles, ZP (*−*24 ± 1.4) was highly negative, and EE (56.26 ± 0.16) was superior to 50%.

### 3.2. Short-Term Stability of Licochalcone-A PLGA NPs

**Figure 3:**
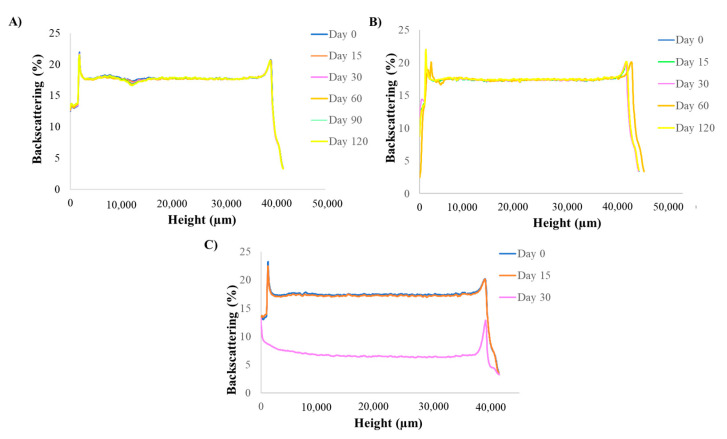
Turbiscan backcattering profile at different temperatures analyzed monthly. (**A**) 4 °C, (**B**) 25 °C and (**C**) 38 °C.

The optimized Lico-A PLGA NPs were stored at three different temperatures (4, 25, and 38 °C) in order to study their short-term stability. As can be observed in Figure 3, Lico-A PLGA NPs were stable at 4 °C and 25 °C even after 3 months of storage. However, at 38 °C, the formulations were clearly unstable obtaining variations of the BS superior to 10% [55,56]. These results correlate with other obtained by previous authors using PLGA nanocarriers [43]. Moreover, differences in the BS profile at 4 and 25 °C were below 10%, which indicates a suitable short-term stability at both temperatures. However, a slight difference after two months of storage can be observed at 25 °C which may indicate an initial instability process, whereas at 4 °C the profiles were almost identical. Therefore, 4 °C will be the most suitable temperature for storing Lico-A PLGA NPs.

### 3.3. Synthesis of CPP and Polymer Conjugation

In order to functionalize Lico-A PLGA NPs, two CPPs were synthesized by solid phase peptide synthesis. A residue of Cys was incorporated at the N-terminal end of both Tet-1 and B6 peptides to perform a covalent linkage with the PLGA-PEG-maleimide copolymer previously synthesized. Peptide characterization by ESI-MS is shown in Table S1 of the supplementary material. The PLGA polymer was activated with NHS and covalently coupled to the maleimide-PEG-NH_2_ by formation of an amide group in liquid phase. Afterwards, conjugation of PLGA-PEG-maleimide with CPP Tet-1 and B6 was carried out in solution, being confirmed by ^1^H-NMR.

### 3.4. Physicochemical Characterization of Licochalcone-A Functionalized Nanoparticles

Table 3 shows the results of the physicochemical properties of surface functionalized Lico-A NPs. It can be observed that both formulations (Lico-A PLGA-PEG-Tet1 NPs and Lico-A PLGA-PEG-B6 NPs) show optimal physicochemical properties for ocular administration. Interestingly, the conjugation of a CPP to the polymer allows to obtain Lico-A NPs with lower Z_av_, probably due to polymer interactions with the positive peptide charge compacting the NPs core. Moreover, CPP conjugation increases the PI in a slight manner, but all the formulations PI remain below 0.2. However, it can be observed that average size and PI of Lico-A PLGA-PEG-Tet1 NPs are slightly higher than Lico-A PLGA-PEG-B6 NPs probably because B6 has a higher positive AA ratio (4 positive AA /9 total AA) than Tet-1 (2 positive AA/12 total AA). Therefore, B6 higher positive charge may favor PLGA interaction, thus contributing to smaller and more homogeneous NPs than Tet-1. In addition, ZP of the obtained peptide targeted NPs showed a surface charge modification from negative to highly positive. This has been previously described by other authors using CPP [8]. Furthermore, this positive charge will ensure better interaction with corneal tissues due to their negative charge [57]. The EE values of Lico-A PLGA-PEG-B6 NPs are significantly lower than Lico-A PLGA NPs and Lico-A PLGA-PEG-Tet-1 NPs where the EE is higher than 50% of the initial drug added. This may be due to the smaller Lico-A PLGA-PEG-B6 NPs Z_av_ that might cause lower EE values [58].

### 3.5. Morphology of Licochalcone-A Functionalized Nanoparticles

**Figure 4:**
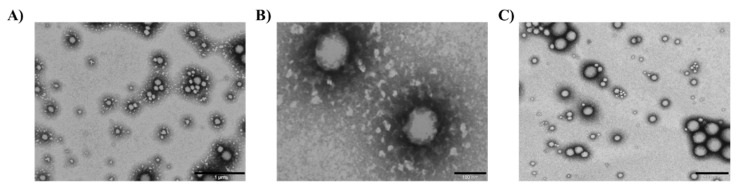
Transmission electron microscopical images of Lico-A NPs. (**A**) Lico-A-PLGA NPs (scale bar corresponds to 1 µm), (**B**) Lico-A PLGA-PEG-Tet-1 NPs (scale bar corresponds to 100 nm), and (**C**) Lico-A PLGA-PEG-B6 NPs (scale bar corresponds to 500 nm).

Lico-A PLGA NPs, Lico-A PLGA-PEG-Tet-1 NPs, and Lico-A PLGA-PEG-B6 NPs were observed by TEM after negative staining (Figure 4). In all the cases, Lico-A loaded NPs showed a spherical shape and a smooth surface, characteristic of this type of drug delivery system, without any aggregation phenomena [52]. As expected, NPs size measured by TEM was similar to the size obtained by PCS [59].

### 3.6. Drug Release of Licochalcone-A Functionalized Nanoparticles

**Figure 5:**
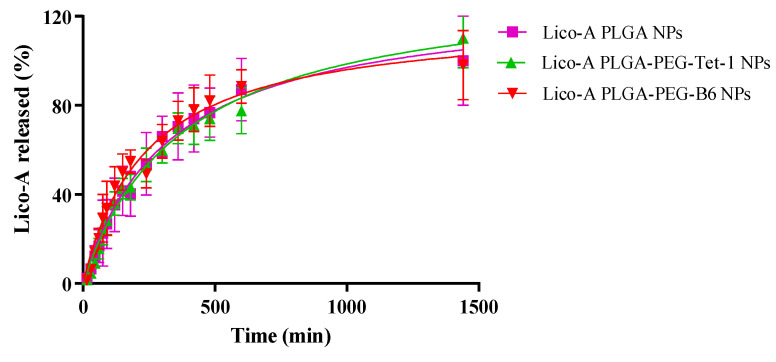
Lico-A release profile carried out by direct dialysis method for studying several formulations (Lico-A PLGA NPs, Lico-A PLGA-PEG-Tet1 NPs, and Lico-A-PLGA-PEG-B6 NPs).

In order to study the release of Lico-A from Lico-A PLGA NPs, Lico-A PLGA-PEG-Tet1 NPs, and Lico-A PLGA-PEG-B6 NPs, a direct dialysis experiment was carried out. In all Lico-A NPs, an initial fast release corresponding to a burst effect of the Lico-A placed on the NPs surface followed by a sustained Lico-A release can be observed (Figure 5).

Moreover, release profiles of all the formulations were very similar, being able to release Lico-A within the first 24 h after their application. In addition, this release from biodegradable nanocarriers is usually governed by a diffusion process since it is much faster than the matrix degradation [55]. Release data were fitted to a hyperbola equation [60,61,62]. Although all of the formulations were very similar, as can be observed in Table 4, Lico-A PLGA-PEG-Tet-1 NPs showed the higher K_d_ (equilibrium dissociation constant) thus meaning that diffusion of Lico-A is slower than the observed in the other formulations, indicating that the formulation obtained with the Tet-1 peptide conjugated to the polymer is able to delay Lico-A delivery. Moreover, in all cases, B_max_ was around 100%, thus indicating that all of the Lico-A encapsulated is released within 24 h [43].

### 3.7. Ocular Tolerance of Licochalcone-A Surface Functionalized Nanoparticles

The potential risk of eye irritation caused by the developed formulations was evaluated by ocular tolerance tests using in vitro and in vivo methods.

**Figure 6:**
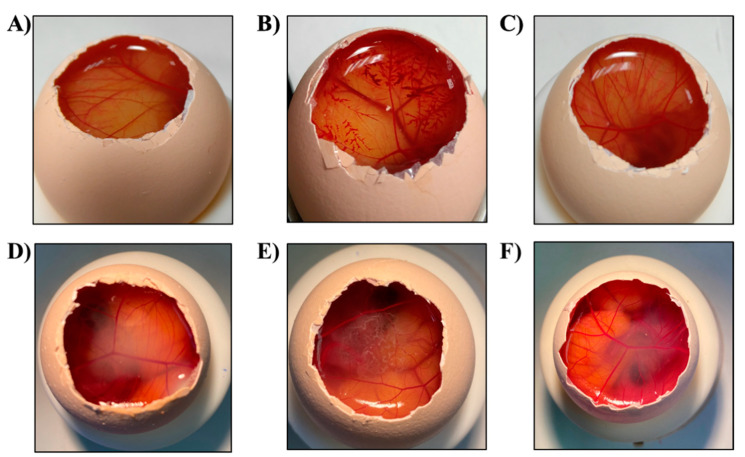
HET-CAM assessment images after 5 min of the product application. (**A**) NaCL, (**B**) NaOH, (**C**) SDS 1%, (**D**) Lico-A PLGA NPs, (**E**) Lico-A PLGA-PEG-Tet-1 NPs, (**F**) Lico-A PLGA-PEG-B6 NPs.

An in vitro HET-CAM test was carried out and OII was calculated. Images were recorded before and after 5 min of the product application to the CAM (Figure 6). Moreover, OII was calculated showing that neither Lico-A PLGA NPs, Lico-A PLGA-PEG Tet-1 NPs, nor Lico-A PLGA-PEG-B6 NPs were irritants. Therefore, all the formulations obtained an OII around 0 without observing any irritation phenomena and they were classified as non-irritant.

However, since the in vitro results are not able to reproduce all of the in vivo conditions, Draize eye irritation of free Lico-A, Lico-A PLGA NPs, Lico-A PLGA-PEG-Tet1 NPs, and Lico-A PLGA-PEG-B6 NPs was carried out to confirm the HET-CAM classification [63].

The results of the in vivo test showed excellent ocular tolerance for all the formulations evaluated (Figure S3). No signs of eye irritation were detected in any of the formulations, being classified as non-irritating substances. Therefore, these results agree with those obtained by the HET-CAM test confirming its suitability for the evaluation of the ocular tolerance of pharmaceutical products [44].

### 3.8. Anti-Inflammatory Therapeutic Efficacy of Licochalcone-A Functionalized Nanoparticles

The anti-inflammatory efficacy of the Lico-A NPs functionalized with Tet-1 or B6 peptides was assessed in a model of ocular inflammation.

**Figure 7:**
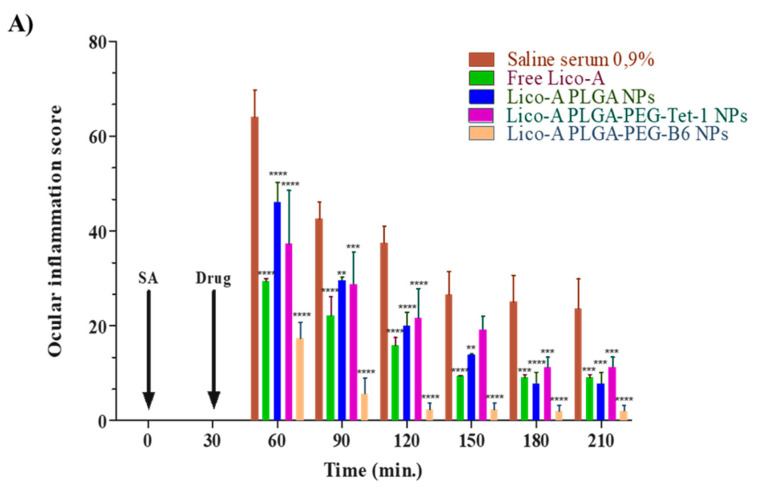

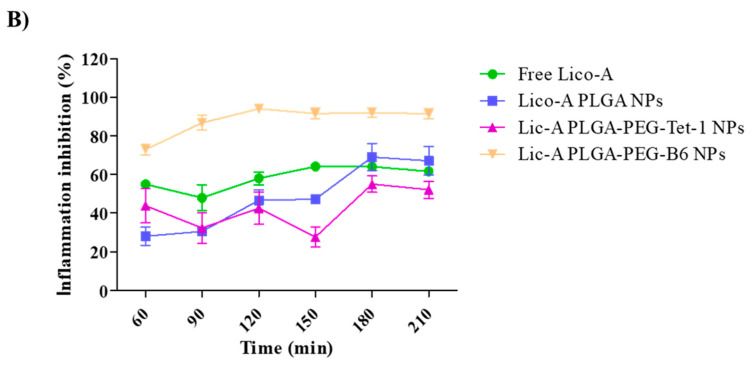
Ocular anti-inflammatory results. (**A**) Ocular inflammation score. (**B**) Inhibition of inflammation (%). Significant differences compared to the saline serum group: ** *p* < 0.01; *** *p* < 0.005; **** *p* < 0.0001.

As can be observed in Figure 6, during the first timepoints, all of the Lico-A NPs and free Lico-A showed statistically significant differences against the control group (*p* < 0.001). This may be due to the potent Lico-A anti-inflammatory capacity previously reported by other groups and demonstrated here for ocular applications [64,65]. Among all the formulations, Lico-A PLGA-PEG-B6 NPs presented significantly increased anti-inflammatory activity compared to the other formulations and free Lico-A (Figure 7). This correlates with the faster initial Lico-A release obtained in vitro as well as with the fact that B6 peptide has a higher positive charge than Tet-1, thus increasing the interactions with negative ocular layers and allowing Lico-A enhanced penetration and therapeutic effects. Furthermore, Lico-A PLGA-PEG-B6 NPs also possess a smaller average size which may facilitate their cellular internalization and pharmacological effects [66]. Moreover, free Lico-A showed an initial superior activity compared to Lico-A PLGA NPs and Lico-A PLGA-PEG-Tet1 NPs but after 90 min no significant differences between the formulations were obtained. Moreover, all of the assessed formulations were able to reduce inflammation compared to the saline serum control in a significant manner, even after 210 min after the inflammation induction.

Therefore, these results demonstrate that Lico-A could be used for ocular inflammation treatment and that surface functionalized NPs were able to decrease inflammation in a more prolonged manner than the free drug. Moreover, Lico-A PLGA-PEG-B6 NPs constitute the best strategy to administer Lico-A in ocular tissues, being able to reduce inflammation in a significantly effective manner.

## 4. Conclusions

In the present manuscript, two formulations of Lico-A loaded peptide-functionalized biodegradable NPs were developed. Moreover, the DoE approach was confirmed to be a suitable method to optimize Lico-A PLGA NPs that showed a suitable short-term stability. In addition, PLGA surface was functionalized with PEG and custom-synthesized CPP obtaining two formulations: Lico-A PLGA-PEG-Tet1 NPs and Lico-A PLGA-PEG-B6 NPs. These formulations were characterized by obtaining an average size below 200 nm and a monodisperse system with highly positive surface charge. In addition, they demonstrated to provide a prolonged Lico-A release, slightly slower for Lico-A PLGA-PEG-Tet1 NPs. In addition, none of the formulations were irritants in vitro or in vivo. Furthermore, the ocular anti-inflammatory efficacy of Lico-A PLGA-PEG-B6 NPs was significantly superior compared to free Lico-A and Lico-A PLGA-PEG-Tet-1 NPs. Despite that, in all the cases the formulations developed showed significant anti-inflammatory effects against the control. However, Lico-A PLGA-PEG-B6 NPs were demonstrated to reduce inflammation in a highly effective manner constituting a promising system to be topically administered for ocular inflammation.

## Figures and Tables

**Table 1:** Matrix of the factorial design.

	Coded Levels				
**Variables**	−1.68	−1.00	0.00	1.00	1.68
Lico-A (mg/mL)	0.16	0.50	1.00	1.50	1.80
PLGA (mg/mL)	4.16	4.50	5.00	5.50	5.84
Tween 80 (%)	0.46	0.60	0.80	1.00	1.14

**Table 2:** Results of the factorial design developed to optimize Lico-A NPs formulation.

Experiment Number	Independent Variables						Dependent Variables			
	Lico-A		PLGA		Tween 80		Z_av_ (nm)	PI	ZP (mV)	EE (%)
	Coded Level	mg/mL	Coded Level	mg/mL	Coded Level	%				
1	−1	0.5	−1	4.5	−1	0.6	130.6	0.103	−39.2	18.80
2	1	1.5	−1	4.5	−1	0.6	144.8	0.098	−36.3	55.43
3	−1	0.5	−1	4.5	1	1.0	126.7	0.091	−35.4	10.38
4	1	1.5	−1	4.5	1	1.0	130.4	0.088	−31.8	19.92
5	−1	0.5	1	5.5	−1	0.6	136.0	0.078	−32,7	30.04
6	1	1.5	1	5.5	−1	0.6	155.0	0.082	−33.4	61.72
7	−1	0.5	1	5.5	1	1.0	166.1	0.103	−33.8	7.02
8	1	1.5	1	5.5	1	1.0	143.5	0.079	−31.6	32.32
9	1.68	1.84	0	5.0	0	0.8	149.6	0.095	−31.7	59.53
10	−1.68	0.16	0	5.0	0	0.8	129.6	0.100	−37.8	35.55
11	0	1.0	0	5.0	1.68	1.136	134.8	0.111	−31.3	8.65
12	0	1.0	0	5.0	−1.68	0.464	135.7	0.085	−33.4	45.46
13	0	1.0	1.68	5.84	0	0.8	139.5	0.088	−35.1	16.25
14	0	1.0	−1.68	4.16	0	0.8	124.4	0.096	−31.6	12.50
15	0	1.0	0	5.0	0	0.8	132.9	0.092	−32.6	13.62
16	0	1.0	0	5.0	0	0.8	128.1	0.099	−30.5	14.14

**Table 3:** Physicochemical parameters of the optimized formulation (1 mg/mL of Lico-A, 8 mg/mL of PLGA, and 0.4%Tween 80) without peptide addition, with PEG and Tet-1 peptide, and with PEG and B6 peptide.

Formulation	Z_av_ (nm) ± SD	PI ± SD	ZP ± SD	EE (%)
Lico-A PLGA NPs	163.81 ± 2.29	0.075 ± 0.010	−24.2 ± 1.4	56.26 ± 0.16
Lico-A PLGA-PEG-Tet-1 NPs	128.65 ± 7.53	0.149 ± 0.016	16.02 ± 0.58	53.26 ± 0.62
Lico-A PLGA-PEG-B6 NPs	114.24 ± 2.42	0.122 ± 0.012	10.49 ± 1.02	31.36 ± 0.60

**Table 4:** Release data fitted to a hyperbola equation (meaning K_d_, half of the time when Lico-A is released at equilibrium and B_max_ maximum % of Lico-A released).

	Lico-A PLGA NPs	Lico-A PLGA-PEG-Tet-1 NPs	Lico-A PLGA-PEG-B6 NPs
B_max_ (%)	128.9 ± 9.3	136.0 ± 10.6	119.3 ± 9.5
K_d_ (min)	328.6 ± 51.4	379.3 ± 61.6	242.0 ± 46.4
Goodness of Fit			
R²	0.8937	0.8891	0.8338

## Data Availability

Data is contained within the article or Supplementary Materials. The data presented of this study are available on request from the corresponding author.

## Supplementary Materials

The following are available online at https://www.mdpi.com/article/10.3390/pharmaceutics14020285/s1, Figure S1: Chemical structure of CPPs. (A) Tet-1 peptide, (B) B6 peptide; Figure S2: Average size DoE analysis. (A) Pareto chart of average size, (B) surface response corresponding to average size obtained with 0.8% of Tween. Figure S3: Ocular tolerance Draize test results after 30 min of the product application. (A) Saline serum (control group), (B) Lico-A PLGA NPs, (C) Lic-A PLGA-PEG-Tet-1 NPs, (D) Lic-A PLGA-PEG-B6 NPs; Table S1. Peptide characterization by ESI-MS.
