# Supplementary material for: Development of Peptide Targeted PLGA-PEGylated Nanoparticles Loading Licochalcone-A for Ocular Inflammation
Source: Pharmaceutics. 2022 Jan 26;14(2):285. doi: 10.3390/pharmaceutics14020285 (PMC8874979; doi:10.3390/pharmaceutics14020285)
Supplement: Supplementary file 1 [file pharmaceutics-14-00285-s001.zip › pharmaceutics-1546037-supplementary.pdf]

pharmaceutics

MDPI

Supplementary Materials: Development of Peptide Targeted PLGA-PEGylated Nanoparticles Loading Licochalcone-A for Ocular Inflammation

Ruth Galindo, Elena Sánchez-López, María José Gómara, Marta Espina, Miren Ettcheto, Amanda Cano, Isabel Haro, Antoni Camins and María Luisa García

A)

B)

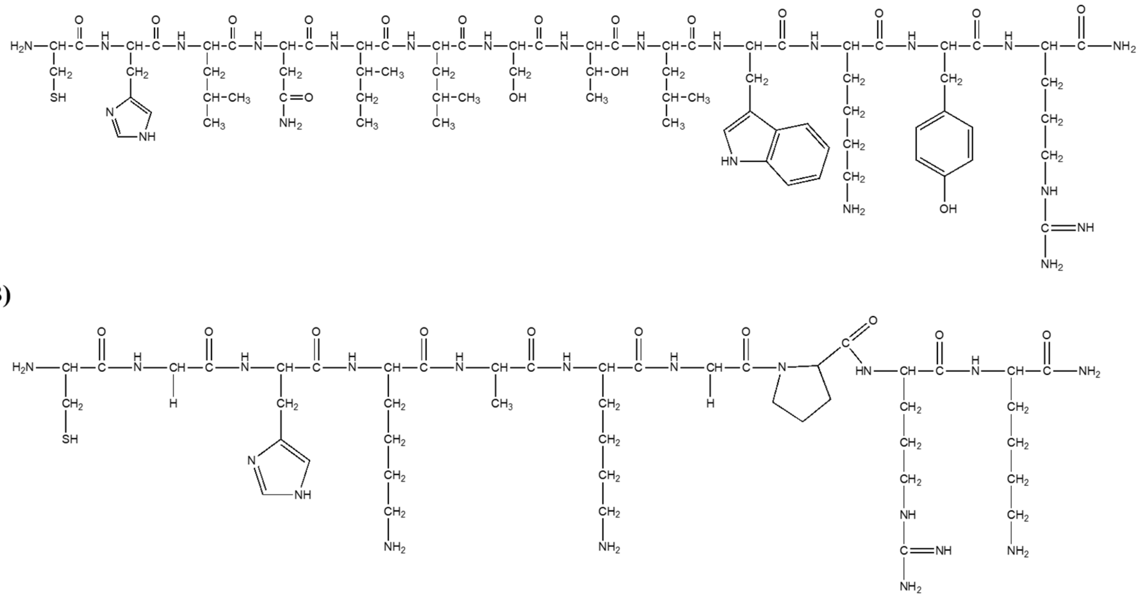

Figure S1. Chemical structure of CPPs. A) Tet-1 peptide, B) B6 peptide.

A) B)

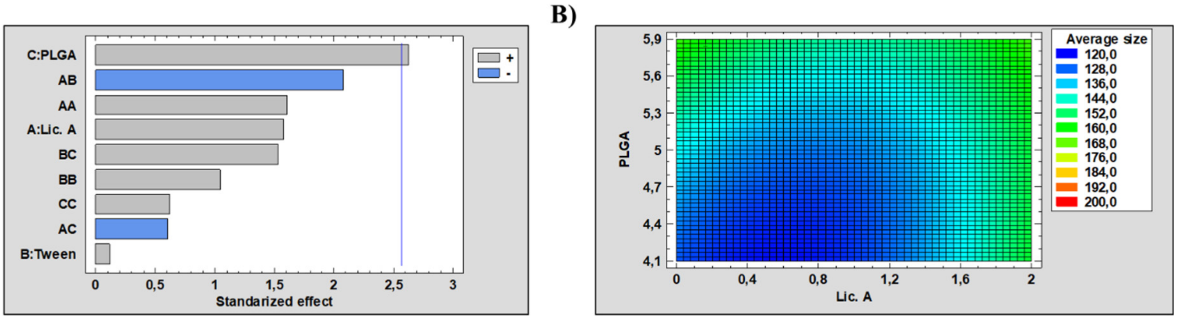

Figure S2. Average size DoE analysis. A) Pareto's chart of average size, B) Surface response corresponding to average size obtained with 0.8 % of Tween.

A) B) C) D)

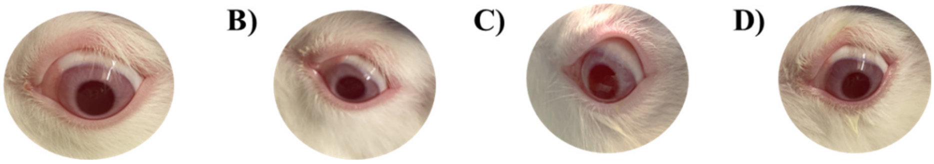

Figure S3. Ocular tolerance Draize test results after 30 min of the product application. A) Saline serum (control group); B) Lico-A PLGA NPs, C) Lic-A PLGA-PEG-Tet-1Cys NPs, D) Lic-A PLGA-PEG-B6 NPs.

Table S1. Peptide characterization by ESI-MS.

	MW (g/mol)	ESI-MS m/z	theoretical m/z	experimental m/z
**Cys-Tet-1** $C_{76}H_{119}N_{21}O_{18}S$	1646.98	$[M+2H]^{2+}$	824.48	823.97
		$[M+3H]^{3+}$	549.99	549.64
		$[M+4H]^{4+}$	412.75	412.73
**Cys-B6** $C_{45}H_{80}N_{18}O_{11}S$	1081.31	$[M+2H]^{2+}$	541.65	541.96
		$[M+3H]^{3+}$	361.44	360.88
